# Efficacy of a long-term pulmonary rehabilitation maintenance program for COPD patients in a real-life setting: a 5-year cohort study

**DOI:** 10.1186/s12931-021-01674-3

**Published:** 2021-03-10

**Authors:** Léo Blervaque, Christian Préfaut, Hélène Forthin, Francis Maffre, Marion Bourrelier, Nelly Héraud, Matthias Catteau, Pascal Pomiès, Dany Jaffuel, Nicolas Molinari, Maurice Hayot, Fares Gouzi

**Affiliations:** 1 PhyMedExp, INSERM U1046, CNRS UMR 9214, Montpellier University, Montpellier, France; 2 Air+R Network, Montpellier University, Montpellier, France; 3 Air+R Network, Montpellier, France; 4 Direction de la recherche clinique et de l’innovation en santé - KORIAN SANTE, 34700 Lodève, France; 5 Department of Pneumology, Montpellier University Hospital, Montpellier, France; 6 IMAG, CNRS, Montpellier University, Montpellier University Hospital, Montpellier, France; 7 PhyMedExp, University of Montpellier, INSERM, CNRS, CHRU, Montpellier, France

**Keywords:** Pulmonary rehabilitation, Maintenance programs, COPD, Survival

## Abstract

**Background:**

Pulmonary rehabilitation (PR) improves exercise capacity, health-related quality of life (HRQoL) and dyspnea in chronic obstructive pulmonary disease (COPD) patients. Maintenance programs can sustain the benefits for 12 to 24 months. Yet, the long-term effects (> 12 months) of pragmatic maintenance programs in real-life settings remain unknown. This prospective cohort study assessed the yearly evolution in the outcomes [6-min walking distance (6MWD), HRQoL, dyspnea] of a supervised self-help PR maintenance program for COPD patients followed for 5 years. The aim was to assess the change in the outcomes and survival probability for 1 to 5 years after PR program discharge in COPD patients following a PR maintenance program supported by supervised self-help associations.

**Methods:**

Data were prospectively collected from 144 COPD patients who followed a pragmatic multidisciplinary PR maintenance program for 1 to 5 years. They were assessed yearly for 6MWD, HRQol (VQ11) and dyspnea (MRC). The 5-year survival probability was compared to that of a control PR group without a maintenance program. A trajectory-based cluster analysis identified the determinants of long-term response.

**Results:**

Maintenance program patients showed significant PR benefits at 4 years for 6MWD and VQ11 and 5 years for MRC. The 5-year survival probability was higher than for PR patients without PR maintenance. Two clusters of response to long-term PR were identified, with responders being the less severe COPD patients.

**Conclusions:**

This study provides evidence of the efficacy of a pragmatic PR maintenance program in a real-life setting for more than 3 years. In contrast to short-term PR, long-term PR maintenance appeared more beneficial in less severe COPD patients.

**Supplementary Information:**

The online version contains supplementary material available at 10.1186/s12931-021-01674-3.

## Introduction

Randomized controlled trials (RCTs) show that short-term pulmonary rehabilitation (PR) programs provide clinically significant improvements in the exercise capacity, health-related quality of life (HRQoL) and dyspnea of COPD patients. The last Cochrane Library meta-analysis concluded that additional RCTs comparing short-term PR to usual care in COPD were no longer warranted and that further studies should focus on new research questions [1]. Currently, a major research question concerns the maintenance of short-term PR benefits 12–24 months after program discharge [2], which is when these benefits are lost [3]. Unfortunately, little is known about strategies to maintain the benefits over years. A few studies have shown that long-term (> 12 months) PR maintenance programs are effective to maintain the short-term PR benefits [4–6]. However, the feasibility of intensive programs outside the research context is problematic, and translating experimental PR maintenance results to clinical settings remains an issue [3]. A feasible PR maintenance program needs to be designed in a real-life setting.

An original and feasible strategy is to base PR maintenance on supervised self-help associations. For example, the French Air + R network has offered supervised PR maintenance programs to patients enrolled in associations within the healthcare system since its creation in 2004 (950 patients to date). We previously showed that the Air + R network maintenance program was clinically effective 12 months after PR discharge [7]. Nevertheless, practitioners need to know that the programs have been carefully evaluated [3], and both medical societies and regulatory authorities have requested the assessment of long-term trajectories and methods to identify responders [8]. Randomizing patients into a usual-care group for many years would not be ethically acceptable, but well-designed observational studies of patient cohorts are adapted to investigating the effectiveness of such healthcare interventions [9, 10]. On this basis, we hypothesized that this program would prevent clinically significant declines in exercise tolerance, HRQoL and dyspnea for at least 3 years. In addition, given COPD patients’ heterogeneous responses to PR [11], we further expected to identify different patterns of response to the program.

Thus, this observational cohort study assessed the yearly change in PR outcomes for 1 to 5 years after PR program discharge in COPD patients following a PR maintenance program supported by a network of supervised self-help associations (“PR + maintenance” group). The primary outcome was the yearly change in 6-min walking distance (6MWD). As secondary outcomes, we assessed: (1) the yearly changes in other PR outcomes (HRQoL, dyspnea), (2) the survival probability at 60 months versus that of a control group (COPD after PR discharge, without a maintenance program: “PR only” group), and (3) the patterns of response to the maintenance program.

## Materials and methods

Detailed information on the maintenance program, outcomes and statistical analysis is provided in Additional file 1.

### Subjects and experimental design

#### COPD patients in self-help associations: “PR + maintenance” group

We conducted a prospective cohort study using a dataset from the French Air + R network created in 2004. The network database contains the yearly evaluations in standardized case-report form of patients who join the network after an initial PR program. The database was screened from January 2011 to December 2018 and data were extracted in December 2018. The inclusion criteria were the following: (1) a COPD diagnosis by a lung specialist based on the association of compatible symptoms (dyspnea, chronic cough or sputum production) and/or a history of exposure to risk factors, as well as a post-bronchodilator forced expiratory volume in 1 s/forced vital capacity ratio < 70% [12]; (2) completion of a short-term inpatient PR program; (3) completion of 1 to 5 years in the maintenance program; and (4) at least two interpretable 6MWD values. For the eligible COPD patients, the “zero-time” point (T0) was set at the maintenance program entry date. Additionally, we collected data on the 6MWD, VQ11 and MRC of these patients, if available, before and after their short-term inpatient PR program. The Institutional Review Board of Montpellier University Hospital approved the study (2018_IRB-MTP_09-02), and informed consent was obtained from each patient. Clinical trial: https://clinicaltrials.gov/ct2/show/NCT03704935.

#### Maintenance program

After a short-term inpatient PR program performed in the East Occitanie region of France, all COPD patients had the opportunity to join the Air + R healthcare network of 12 local self-help patient associations (for further information, please see https://www.airplusr.fr) in the following weeks. This network offers multidisciplinary PR maintenance programs comprising: (1) individualized exercise training (~ 42 sessions/year), (2) health education classes (6 h/session; 2 sessions), and (3) psychological support (~ 2.5 sessions/year). Financial support to the self-help associations for these supervised activities is provided by the Air + R healthcare network.

#### “PR only” group

For survival analysis, we extracted data from the database of the two main PR centers in the study region, both of which are recruitment sources for the Air + R healthcare network (Cliniques du Souffle La Vallonie and la Solane). The inclusion criteria were similar as those of the “PR + maintenance” group (i.e., spirometry-based COPD diagnosis by the referent chest physician and completion of a short-term inpatient PR program), except that these patients did not participate in a maintenance program after completion of the short-term PR program. The database was screened for the same period (from January 2011 to December 2018) and for patients who lived in the same region as the patients in the “PR + maintenance” group (i.e., East Occitanie, France). After database screening, a random subset of eligible patients was obtained. Last, to avoid overlap between the “PR-only" group and the “PR + maintenance” group, the databases of the Air + R network and the PR centers were compared, and patients present in both were excluded. Thus, for this survival analysis, the T0′ point for the eligible COPD patients was set at the end of the short-term impatient PR program. The data from patients with a follow-up longer than 60 months or without events up to 31 December 2018 were right-censored.

### Outcomes

Patients in the maintenance program were evaluated for baseline values of all parameters at inclusion (T0) and then every 12 months for a maximum of 60 months (T12–T60).

#### Primary outcome measure

6MWD was assessed following international recommendations [13]. Briefly, patients were asked to cover the longest possible distance along a 30-m corridor. Tests were performed twice to control the learning effect. The predicted 6MWD value was determined using the equation of Troosters et al. [14].

#### Secondary outcome measures

##### Health-related quality of life

The VQ11 questionnaire assessed health-related quality of life. This short self-administered questionnaire was validated in COPD patients [15, 16].

##### Dyspnea

The impact of dyspnea was assessed with the Medical Research Council (MRC) dyspnea scale, which was validated and has prognostic value for COPD patients [17].

##### Lung function and BODE index

Lung function was assessed by a lung specialist using a plethysmographic spirometer and recorded in the network database. The BODE index was calculated as previously described [18].

##### Survival analysis

To compare the survival probability of COPD patients from the “PR + maintenance” group with that of the “PR only” group, we extracted the vital status of these patients from the *Institut national de la statistique et des études économiques* (INSEE) database. This database includes all deaths of French people from 1977 to January 2020, with information on name, date and place of birth, and date and place of death. To assess the deaths among patients from our databases, we performed automatic matching with the INSEE database based on date of birth and the first 10 digits of the social security number. To avoid misclassification, we performed two other matching methods, one with a combination of name and the first 10 numbers of the social security number and another with a combination of name and date of birth. The total number of deaths within each group was determined after rigorous comparison of these three datasets.

##### Statistical analysis

The evolution of the primary and secondary outcomes during follow-up was assessed using linear mixed effect models (LME) fit with the *nlme* R package [19], including a Time effect as fixed effect and a Subject effect as random effect. Assumptions of normal distribution of the residuals and homoscedasticity were graphically verified for each LME test. No multiple imputation method has been performed. False discovery rate (FDR)-adjusted post-hoc analysis was performed in order to restrict the number of false discoveries due to the multiplicity of the tests. In particular, given the long follow-up period, analyses were adjusted on the age effect when age was significant. Survival analysis from T0′ was performed by producing Kaplan–Meier survival curves and COX uni- and multivariate regression analyses, fitted with the *survival* and *survminer* R packages [20, 21] and allowing calculations of the adjusted hazard ratio (HR). Proportional hazards has been graphically checked. Right censoring was applied for patients who joined the program after 2013 and had not completed all assessments at the time of data extraction (December 2018). Trajectories were analyzed using latent class mixed models fitted with the *lcmm* R package [22]. A p-value < 0.05 was considered significant. All analysis was performed using R 3.5.0 software (www.r-project.org).

## Results

### Patient characteristics and maintenance program adherence

**Fig. 1:**
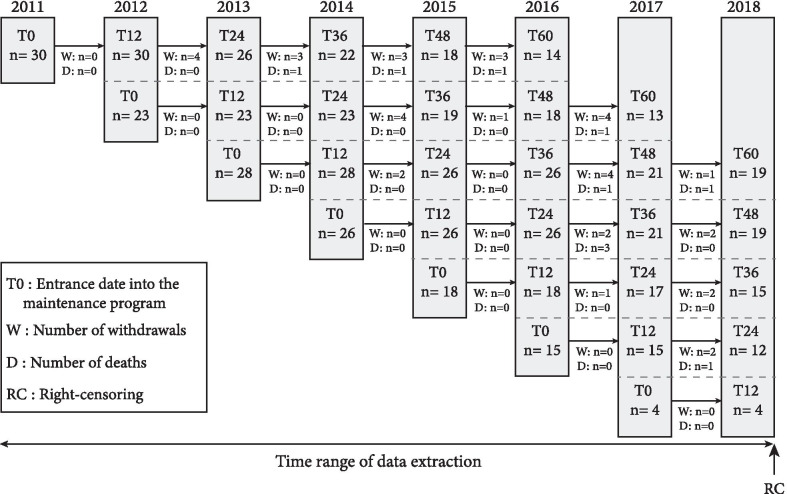
Flow chart of subject participation for the “PR + maintenance” COPD patients. *COPD* chronic obstructive pulmonary disease. Right-censored data are from patients who did not perform all evaluations due to program entry after 2013 and not due to withdrawal (e.g., patients joining the program in 2015 could only be evaluated up to T36, the data extraction and analysis having been done in December 2018)

During the study period, 144 COPD patients were eligible. Baseline characteristics of the “PR + maintenance” patients included in the analysis are fully detailed in Table 1. Most patients (n = 105, 73%) had complete survival follow-up to death or censoring on 31 December 2018. Figure 1 presents the design of the patient inclusion procedure. From T0, the mean follow-up duration was 41.6 ± 16.4 months. The dropout rate was 27% over the 5 years, with 39 patients withdrawing and 10 patients who died. The main reasons for withdrawal were clinical worsening (n = 10), motivation (n = 7), logistics (n = 6), relationships within the network (n = 6), and others (n = 10). Withdrawn patients did not differ from other patients in terms of severity (BODE index: 2.30 ± 1.94 vs*.* 2.75 ± 1.86; p = 0.28), exercise tolerance (6MWD: 71.23 ± 17.87% vs*.* 75.59 ± 16.70%; p = 0.21), dyspnea (MRC: 2.38 ± 1.29 vs*.* 2.48 ± 1.25; p = 0.68) or quality of life (VQ11: 23.90 ± 7.58 vs*.* 26.11 ± 8.48; p = 0.21). Some patients who joined the program after 2013 had not completed all the assessments in December 2018. Thus, they were right-censored (RC) at that time (Fig. 1). The rate of adherence to the maintenance program was 53 ± 22% (sessions attended/sessions scheduled). Over the 5 years, only one adverse event related to the maintenance program was reported (knee synovial effusion).

**Table 1:** Baseline characteristics of the “PR + maintenance” COPD patients

	n = 144
Sex ratio (%*males)*	56.9%
Age *years*	66.26 (8.48)
BMI *kg/m*^*2*^	26.59 (5.29)
FEV_1_ *%pred*	63.18 (25.78)
FEV_1_/VC	52.73 (13.24)
Disease severity (GOLD)	
I	25.6%
II	40.6%
III	24.8%
IV	9.0%
Smoking history *pack-year*	37.89 (25.81)
BODE Index	2.64 (1.88)
6MWD *m*	454.51 (97.98)
6MWD *%pred*	74.38 (17.07)
MRC	2.45 (1.26)
VQ11	25.53 (8.28)
Comorbidities	
Mean number of comorbidities per patient	2.26 (1.72)
Pulmonary *n (% total)*	66 (45.8%)
Cardiovascular *n (% total)*	86 (59.7%)
Metabolic *n (% total)*	47 (32.6%)
Neurologic *n (% total)*	7 (4.9%)
Joint and muscle disorders *n (% total)*	16 (11.1%)
Others *n (% total)*	23 (16.0%)

Data are presented as means (SD). Disease severity classified according to the GOLD guidelines: stage I, mild, FEV1 > 80% of predicted normal value; stage II, moderate, FEV1 50–79%; stage III, severe, FEV1 30–49%; stage IV, very severe, FEV1 < 30%

*BMI* body mass index, *FEV1* forced expiratory volume in 1 s, *VC* vital capacity, *6MWD* 6-min walking distance, *%pred* % predicted, *MRC* modified Medical Research Council dyspnea score, *VQ11* short health-related quality of life questionnaire

### Effect of the maintenance program

**Fig. 2:**
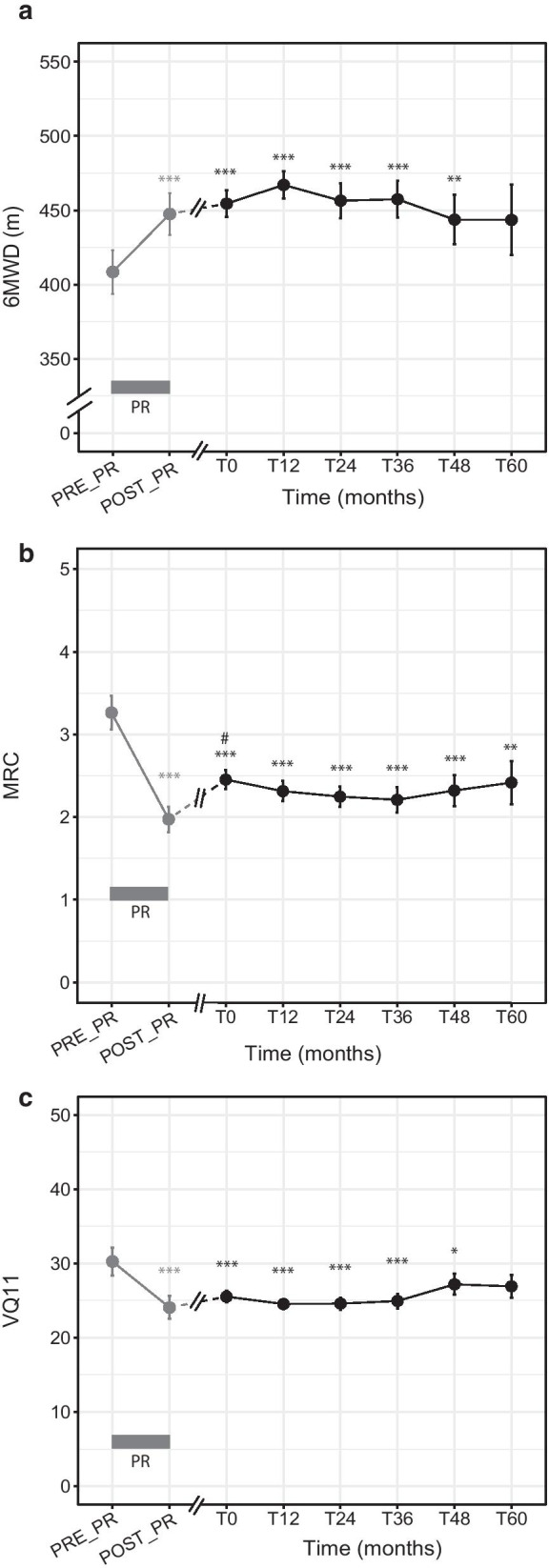
Pattern of change in pulmonary rehabilitation outcomes over time in the “PR + maintenance” group. **a** Change in 6-min walking distance (6MWD). **b** Change in Medical Research Council (MRC) dyspnea score. **c** Change in health-related quality of life score (VQ11). Gray box: pulmonary rehabilitation (PR) program. Gray dots and lines: pre- and post-PR values. Black dots and lines: values during the maintenance program. Linear mixed effects model. Time effect: p < 0.001 for (**a**–**c**). Post-hoc: different from pre-PR: *p < 0.05; **p < 0.01; ***p < 0.001. Different from post-PR: ^#^p < 0.05

The evolutions in outcome variables over time are presented in Fig. 2. Figure 2a shows that the COPD patients in the maintenance program presented no significant difference between 6MWD at program entry (T0) and at any point in the 5-year follow-up (T12, T24, T36, T48, T60). Results were the same for MRC (Fig. 2b) and VQ11 (Fig. 2c). Pre- and post-PR 6MWD values were obtained for 38% (n = 55) of these patients. This subgroup did not differ from the total population of maintenance program patients in age, sex ratio, disease severity, BODE index, 6MWD, MRC or VQ11 (Additional file 1: Table S1). Moreover, the change in 6MWD over the 5 years did not differ between this subgroup and patients without PR data (group × time interaction: p = 0.68; data not shown). In patients with these data, we found that the significant effect of the initial PR remained up to month 48 for 6MWD and VQ11 (Fig. 2a, c) and month 60 for MRC (Fig. 2b). In all “PR + maintenance” patients, 0.26 ± 0.59 unprogrammed medical consultations per year and 0.86 ± 4.74 hospitalization days per year were recorded during the 5-year follow-up.

### Survival analysis

**Fig. 3.**
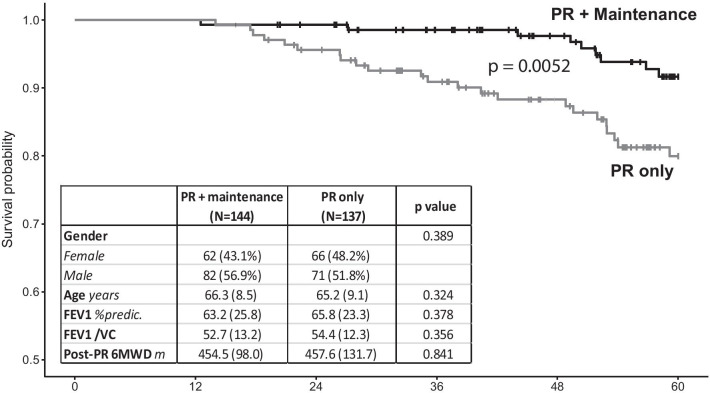
5-year survival probability for the “PR + maintenance” and “PR only” groups. Curves: Kaplan–Meier analysis; gray line: “PR only” group; black line: “PR + maintenance” group. Table: Comparison of main clinical characteristics of the “PR + maintenance” and “PR only” groups

During the study period, 137 COPD patients were eligible for the “PR only” group. Baseline characteristics at T0′ of these patients did not significantly differ from those of the “PR + maintenance” patients (Fig. 3). In addition, 6MWD at T0′ did not differ between the two groups (“PR only”: 458 ± 132 m vs*. “*PR + maintenance”: 454 ± 98 m; p = 0.84; Fig. 3). The mean follow-up duration did not differ between groups (“PR only”: 50 ± 13 vs*.* “PR + maintenance”: 52 ± 12 months; p = 0.14). Kaplan–Meier analysis showed that the survival probability at 60 months was significantly higher in the “PR + maintenance” group compared to “PR only” (log-rank: p = 0.005; Fig. 3). Multivariate regression analysis including statistically significant cofounders (sex, FEV_1_/VC and post-PR 6MWD) confirmed that the adjusted risk of mortality in the “PR + maintenance” patients was significantly and 3.1 times lower than that of “PR only” patients (95% CI 1.0–9.6; p = 0.045; Additional file 1: Table S2).

### Trajectory models

**Fig. 4:**
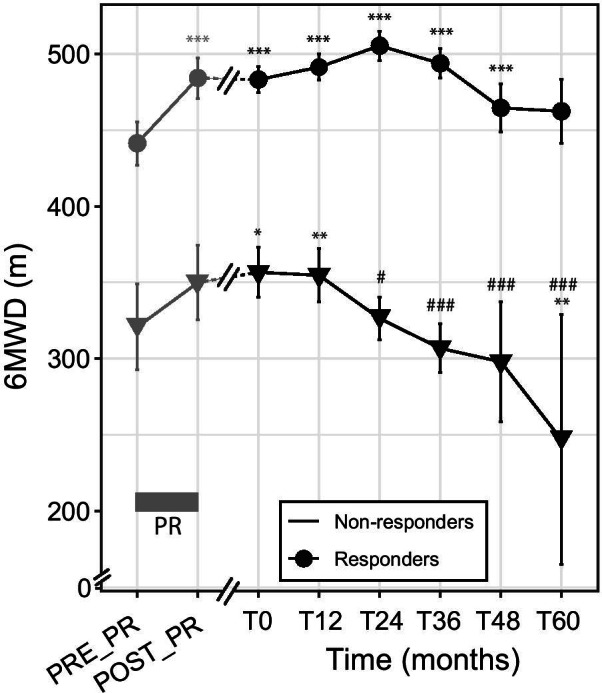
Two-class model showing the mean trajectory of the primary outcome (6-min walking distance) over 5 years of follow-up. PR: pulmonary rehabilitation. Gray box: pulmonary rehabilitation (PR) program. Gray dots and lines: pre- and post-PR values. Black dots and lines: values during the maintenance program. Linear mixed effects model: group × time: group/time interaction effect, p < 0.001. Post-hoc: different from pre-PR: *p < 0.05; **p < 0.01; ***p < 0.001. Different from T0: ^#^p < 0.05; ^###^p < 0.001

To identify patterns of response to the maintenance program, we performed latent class trajectory models. Two-, three- and four-class models were tested. The two-class model presented the best combination of fit indices (lowest Bayesian information criterion and highest entropy) and was thus selected. We identified one cluster of responder patients and one cluster of non-responders. The non-responder cluster included 21% of the maintenance program patients (n = 30). Figure 4 presents the evolution of the primary outcome, 6MWD, along the 5-year follow-up for the two clusters, which presented significantly different evolutions (group × time interaction: p < 0.001; Fig. 4). The 6MWD values of the non-responder cluster significantly declined from month 24 onward (Fig. 4). Conversely, the responder cluster showed no significant 6MWD decline over the follow-up (Fig. 4), and the benefits of the initial PR persisted significantly up to month 48 (Fig. 4). Baseline characteristics of the two clusters are presented in Table 2. The responder cluster had less severe COPD than the non-responder cluster, significantly higher baseline HRQoL and exercise tolerance, and lower dyspnea (Table 2). This cluster more frequently presented at least one comorbidity and was more prone to pulmonary comorbidities and joint disorders (Table 2). Program adherence tended to be higher in the responder cluster (Additional file 2: Fig. S1A, p = 0.07), whereas the initial response to PR did not differ between the two clusters (Additional file 2: Fig. S1B).

**Table 2:** Baseline characteristics of the “PR + maintenance” COPD patients by trajectory class

	Non-responders (N = 30)	Responders (N = 114)	p value
Sex ratio (%*males)*	80.0%	50.9%	0.004
Age *years*	67.83 (10.21)	65.84 (7.97)	0.254
BMI *kg/m*	25.87 (5.20)	26.80 (5.32)	0.416
FEV_1_ *%pred*	50.79 (23.66)	66.20 (25.48)	0.006
FEV_1_/VC	46.74 (12.47)	54.47 (13.03)	0.028
Disease severity (GOLD)			0.022
I	11.5%	29.0%	
II	38.5%	41.1%	
III	26.9%	24.3%	
IV	23.1%	5.6%	
Smoking history *pack-year*	51.48 (27.32)	34.64 (24.55)	0.018
BODE Index	4.55 (1.77)	2.15 (1.58)	< 0.001
6MWD *m*	356.70 (87.13)	483.04 (81.43)	< 0.001
6MWD *%pred*	55.44 (12.53)	79.97 (13.96)	< 0.001
MRC	3.46 (1.21)	2.16 (1.12)	< 0.001
VQ11	29.83 (7.97)	24.40 (8.02)	0.004
Comorbidities *n (% total)*			
Mean number of comorbidities per patient	1.70 (2.14)	2.41 (1.57)	0.043
Pulmonary	7 (23.3%)	59 (51.8%)	0.005
Cardiovascular	14 (46.7%)	72 (63.2%)	0.101
Metabolic	7 (23.3%)	40 (35.1%)	0.222
Neurologic	1 (3.3%)	6 (5.3%)	0.662
Joint disorders	0 (0.0%)	16 (14.0%)	0.030
Others	2 (6.7%)	21 (18.4%)	0.118

Data are presented as means (SD). Disease severity classified according to the GOLD guidelines: stage I, mild, FEV1 > 80% of predicted normal value; stage II, moderate, FEV1 50–79%; stage III, severe, FEV1 30–49%; stage IV, very severe, FEV1 < 30%

*BMI* body mass index, *FEV1* forced expiratory volume in 1 s, *VC* vital capacity, *6MWD* 6-min walking distance, *%pred* % predicted, *MRC* modified Medical Research Council dyspnea score, *VQ11* a short health-related quality of life questionnaire

## Discussion

This long-term follow-up study is the first to show the time course of major PR outcomes for more than 3 years in patients included in a PR maintenance program in a real-life setting. The outcome values during follow-up showed sustained benefits for 48 to 60 months.

The COPD patients in the maintenance program were evaluated at study entry (T0) for 6MWD, quality of life and dyspnea. We then showed that these T0 values did not significantly decline over the 5-year follow-up (Fig. 2). However, the lack of statistically significant declines did not allow us to confidently conclude that the clinical outcomes were stabilized. Thus, to confirm the long-term program efficacy, we tested its effect by comparing values over the course of the maintenance program with the values before the short-term PR in a representative patient subgroup (Additional file 1: Table S1). After significant improvements in exercise tolerance, HRQoL and dyspnea induced by short-term PR (as reported by Lacasse et al. [23]), these maintenance-program patients were able to maintain these significant improvements for 4 years for exercise tolerance and quality of life (Fig. 2a, c) and 5 years for dyspnea (Fig. 2b). In contrast, an earlier study reported that COPD patients were unable to maintain significant PR benefits 12 months after PR discharge without maintenance strategies [2]. An alternative maintenance strategy consisting of repeated yearly short-term PR programs was proposed [24], but the improvements were no longer significant 12 months after PR discharge and provided inconstant benefits for elevated healthcare cost. Here we provide evidence that a pragmatic maintenance program supported by self-help associations offers a reliable response to the challenges of maintaining short-term PR benefits.

As 6MWD and dyspnea have been associated with survival probability in COPD patients [25, 26], we also investigated whether the patients in the Air + R maintenance program presented a survival advantage versus the control “PR only” group, which was composed of COPD patients who differed from our “PR + maintenance group” only by the lack of a maintenance program during follow-up. The external validity of this “PR only” group was acceptable because the mortality at 48 months was 88%, in line with previous studies reporting a 4-year survival probability of 85% in COPD patients with similar exercise capacity (post-PR 6MWD > 305 m) [27]. From T0′ (i.e., the end of the short-term inpatient PR program), the “PR + maintenance” patients had a better prognosis compared to the “PR only” group during the 60 months of follow-up, with an adjusted risk of mortality 3.1 times lower. In order to strengthen this observational result, we considered every confounding factor in a multivariate analysis (Additional file 1: Table S2), which confirmed the increased survival in these patients. The observed survival also appeared higher than in other published “PR only” groups. While the survival probability of our “PR + maintenance” group reached 92% at 60 months, Camillo et al. reported survival probabilities ranging from 81 to 69% at 60 months in COPD patients with a similar exercise capacity. Although our result will have to be confirmed by other studies using different methodologies, it is clearly an additional argument for broader thinking in medical decision-making and healthcare policies regarding pulmonary rehabilitation maintenance strategies [9, 28, 29].

In line with studies that have isolated the phenotype of patients who respond to PR [11], we identified trajectories of response to our maintenance program. Whereas Soicher et al. [30] identified trajectory clusters following a single PR, our study is the first to identify responses to a long-term PR maintenance program, classed into two clusters: a cluster of responders and a cluster of non-responders. Fortunately, only 21% of the patients were non-responders (Table 2), indicating that this program was efficient in prolonging the benefits of the initial PR in a large majority of patients. A previous study showed heterogeneous responses to short-term PR, with a better response for patients with the highest BODE index scores [11]. In contrast, in our study it was the non-responding group that comprised the most severe COPD patients with the highest BODE scores (Table 2). Nevertheless, it should be noted that long-term maintenance and short-term PR programs differ in terms of intensity, frequency and duration. Thus, it is not surprising that short-term and long-term PR were related to different determinants. In line with the previous study [11], we also observed that the short-term PR response was higher in patients with reduced 6MWD (Additional file 3: Fig. S2). Furthermore, the response to the short-term PR did not differ between the responders and non-responders of the maintenance program (Additional file 2: Fig. S1). Altogether, our study is the first to provide a clear picture of the COPD patients who benefit from a PR maintenance program. This may be of great interest for healthcare providers who must define the PR organization for their patients. Given the specific determinants of the response to a long-term maintenance program, this study also opens new avenues for research on the responses to PR maintenance strategies.

The long-term efficacy and determinants of response to the maintenance program may have been mediated by the Air + R network’s self-help associations. This maintenance program has been operating in a clinical context for 15 years and more than 950 patients have benefited from its experience. It is based on self-help communities, which are known to promote long-term exercise adherence [31, 32]. Community-based programs are of growing interest in the field of pulmonary rehabilitation [33]. A recent review identified facilitators and barriers to physical activity maintenance after PR discharge [34]. The supervised sessions and health education in the maintenance program ensured continued support from healthcare professionals, who are key facilitators, and circumvented the key barriers: lack of positive feedback and lack of structured sessions [34]. In addition, the self-help organization and discussion groups provided peer interactions, self-monitoring and routines, which are also key facilitators [34]. These factors together may explain the mean program adherence of over 50% despite the long-term nature of the intervention. Moreover, the dropout rate at month 36 was lower than the dropout rate of the study with the longest follow-up in this context [6]. Last, the psychological and social support from the self-help association and the group dynamics may also have contributed to the PR maintenance program’s efficacy, as suggested [35].

### Study limitations

A limitation for the survival analysis of this study was the non-randomized design. However, several RCTs have shown the efficacy of experimental maintenance programs up to 36 months [2, 6, 7], and we thus assumed that randomizing COPD patients into a group without a maintenance program would be unethical. Conversely, observational studies and RCTs agree head-to-head, if they are well-designed [9, 29, 36]. In the present study, the inclusion criteria were predefined; the patients of both groups were recruited at the same time and place and did not differ in terms of major disease characteristics. Also, the outcomes were prospectively recorded from a definite “zero-time” point in each group and over a follow-up duration that was not statistically different between groups. Nevertheless, a self-selection risk could still be related to the mode of entry into the maintenance program. Indeed, patients with the highest level of self-efficacy or response to the initial PR could constitute the ones who agreed to continue the rehabilitation and attend a maintenance program. In addition, social isolation—which is a barrier to physical activity after a PR [34]—could be unequally distributed among the two groups. Given that these factors are known severity or prognosis factors in COPD [37, 38], this factors could constitute potential biases of the survival analysis. Thus, we strengthened the results as much as possible with statistical adjustments based on multivariate analysis. Last, studies have demonstrated the limits of RCTs in providing evidence for medical decisions and have emphasized the value of real-life observational cohorts [9, 10, 29]. Altogether, the present study presents robust data regarding the long-term efficacy of a PR maintenance program in a real-life setting.

Another limitation was the failure to obtain the initial PR data for all patients, although the subgroup of patients with PR data was representative of the whole COPD group included in the study (Additional file 1: Table S1). Also, the primary outcome, i.e., the evolution of 6MWD, did not differ between patients with and without PR data.

## Conclusion

Based on a well-designed observational study, our results showed that a pragmatic, supervised, self-help PR maintenance program can extend the benefits of the initial PR for more than 3 years (up to 4 years for exercise capacity and health-related quality of life and up to 5 years for dyspnea) in a real-life setting. In addition, this longest PR maintenance follow-up study to date demonstrated favorable survival indicators compared to a comparable control group without maintenance intervention. We identified a small proportion of patients who did not respond to the program. In contrast to the findings for short-term PR, long-term responders were the less severe COPD patients. Although complementary research studies with other designs are warranted, this study adds valuable information regarding the indications and decisions for pulmonary rehabilitation.

## Supplementary Information

**Additional file 1.** Additional Tables, materials and methods.

**Additional file 2: Fig. S1. ** Comparison of maintenance program adherence and response to initial pulmonary rehabilitation program between PR maintenance program responders and non-responders. A. Maintenance program adherence in non-responders (gray bar) and responders (black bar). B. Post-to-pre-pulmonary rehabilitation delta of 6MWD values in non-responders (gray bar) and responders (black bar).

**Additional file 3: Fig. S2.** Relation between the 6-min walking distance (6MWD) at program entrance and the response to this program for short-term PR and long-term PR maintenance program. Short-term: Responders were COPD patients with a gain of 6MWD > 35 m after PR completion. Long-term: Responder and non-responder groups are set on the basis of trajectory analysis described on Fig. 4 and Table 2.

## Data Availability

Data are available upon reasonable request.
