# Supplementary material for: Efficacy of a long-term pulmonary rehabilitation maintenance program for COPD patients in a real-life setting: a 5-year cohort study
Source: Respir Res. 2021 Mar 10;22:79. doi: 10.1186/s12931-021-01674-3 (PMC7948332; doi:10.1186/s12931-021-01674-3)
Supplement: Supplementary file 1 — Additional file 1. Additional Tables, materials and methods. [file 12931_2021_1674_MOESM1_ESM.docx]

Efficacy of a long-term pulmonary rehabilitation maintenance program for COPD patients in real-life setting – A 5 years cohort study

Léo Blervaque^1*^, Christian Préfaut^2^, Hélène Forthin^2^, Francis Maffre^2^, Marion Bourrelier^2^, Nelly Héraud^3^, Matthias Catteau^1^, Pascal Pomiès^1^, Nicolas Molinari^4^, Maurice Hayot^5^, Fares Gouzi^5^

# Additional material & methods

## Subjects and experimental design

### “PR only” group

For survival analysis, we extracted data from a database of patients who participated in a short-term PR program during the same period (2011 to 2017) and in the same region (Occitanie Est, France) as the PR+maintenance group. As the first health status was available at 12 months in the “PR+maintenance” group, patients of the “PR only” group who died during the first 12 months following the PR program were excluded from the study.

## PR maintenance program

COPD patients had the opportunity to join a regional healthcare network of 12 local self-help patient associations: Air+R (for further information please see [www.airplusr.fr](http://www.airplusr.fr)). To do so, they had to have recently completed (within a few months) a certified inpatient PR program conducted along international guidelines (1). This network offers multidisciplinary PR maintenance programs comprising: (1) individualized exercise training with endurance sessions targeting the first ventilatory threshold (outdoor and indoor walking) and interval, upper limb and resistance/strength sessions (local gym), all supervised by an adapted physical activity teacher once a week outside of school breaks (~42 sessions/year); (2) health education classes (6 hr/session; 2 sessions/year in a local conference room) with multidisciplinary health professionals from the network; and (3) psychological support via discussion groups supervised by a psychologist (~2.5 sessions/year). Financial support to the self-help associations for these supervised activities was provided by the Air+R healthcare network.

## Assessments

Patients were evaluated for baseline values of all parameters at network entry, which averaged 86 days after PR completion (T0), and then every 12 months for a maximum of 60 months (T12-T60). As this network includes 12 associations, its research committee, a multidisciplinary team of physicians and scientists, standardized the evaluations and the protocol before the start of the study.

#### Health-related quality of life

The VQ11 questionnaire assessed health-related quality of life, which showed good measurement properties and provided a valid and reliable measure of COPD-specific HRQoL. This short self-administered questionnaire has been validated by our group in COPD patients in French and English language (2, 3) and is used in routine care. It is composed of 11 items (1-5 points/item) along three dimensions: (1) functional, three items; (2) psychological, four items; and (3) social, four items. Good quality of life is characterized by a low VQ11 score. A VQ11 score ≥2 reflects poor quality of life.

#### Healthcare resource utilization

The utilization of healthcare resources was assessed through the yearly number of hospitalization days and the number of unprogrammed consultations during the PR maintenance program.

#### Program adherence

Adherence to the PR maintenance program was assessed by the multidisciplinary team for each activity. The number of attended sessions was recorded for each patient and compared to the number of scheduled sessions.

#### BODE index

The BODE index is calculated from body mass index (B), degree of airflow obstruction: FEV_1_ (O), dyspnea score: MRC (D), and exercise capacity: 6MWD (E). This index is a 10-point scale; the higher the score is, the higher the risk of death (4).

## Statistical analysis

The evolution of the primary and secondary outcomes during follow-up was assessed using linear mixed effect models (LME) fit with the *nlme* R package (5), including a Time effect as fixed effect and a Subject effect as random effect. Given the long follow-up period, analyses were adjusted on the age effect when age was significant. When the Time effect was significant, post-hoc tests corrected by the false discovery rate method were performed to compare each evaluation to baseline.

Trajectories were analyzed using latent class mixed models fitted with the *lcmm* R package (6). Different numbers of clusters were tested and selected based on the Bayesian information criterion (BIC) and entropy. Patients’ baseline characteristics from clusters 1 and 2 were compared using t-, Mann-Whitney or Chi² tests, depending on the data type. Outcome measures were compared using LME including a Time effect, a Group effect, and the interaction between these effects as fixed effects and a Subject effect as random effect. A p-value <0.05 was considered significant. All analysis was performed using R 3.5.0 software [(www.r-project.org).](https://www.r-project.org/)

# References

1. Spruit MA, Singh SJ, Garvey C, Zu Wallack R, Nici L, Rochester C, Hill K, Holland AE, Lareau SC, Man WDC, Pitta F, Sewell L, Raskin J, Bourbeau J, Crouch R, Franssen FME, Casaburi R, Vercoulen JH, Vogiatzis I, Gosselink R, Clini EM, Effing TW, Maltais F, Van Der Palen J, Troosters T, Janssen DJA, Collins E, Garcia-Aymerich J, Brooks D, *et al.* An official American thoracic society/European respiratory society statement: Key concepts and advances in pulmonary rehabilitation. *Am J Respir Crit Care Med* 2013;188:e13-64.

2. Ninot G, Soyez F, Préfaut C. A short questionnaire for the assessment of quality of life in patients with chronic obstructive pulmonary disease: Psychometric properties of VQ11. *Health Qual Life Outcomes* 2013;11:179.

3. Ninot G, Soyez F, Fiocco S, Nassih K, Morin AJS, Prefaut C. Le VQ11, un questionnaire de qualité de vie spécifique à la BPCO utilisable en clinique. *Rev Mal Respir* 2010;27:472–481.

4. Celli BR, Cote CG, Marin JM, Casanova C, Montes de Oca M, Mendez RA, Pinto Plata V, Cabral HJ. The Body-Mass Index, Airflow Obstruction, Dyspnea, and Exercise Capacity Index in Chronic Obstructive Pulmonary Disease. *N Engl J Med* 2004;350:1005–1012.

5. Pinheiro J, Bates D, DebRoy S, Sarkar D. R Development Core Team. 2014. nlme: linear and nonlinear mixed effects models. R package version 3.1-117. *R Found Stat Comput Vienna, Austria* 2014;at <http://cran.r-project.org/package=nlme>.

6. Proust-Lima C, Philipps V, Liquet B. *Estimation of Extended Mixed Models Using Latent Classes and Latent Processes: The* R *Package* ***lcmm***. *J Stat Softw* 2017.

# Additional tables

| **Table S1**. Baseline characteristics of subgroups with or without PR data. | | | | | | |
| --- | --- | --- | --- | --- | --- | --- |
|  | | **Subgroup without PR data (N=89)** | | **Subgroup with PR data (N=55)** | | **p value** |
| **Sex ratio** *M/F* | 55.1% | | 60.0% | | 0.560 | |
| **Age** *years* | 66.16 (9.11) | | 66.42 (7.43) | | 0.858 | |
| **BMI** *kg.m^-^²* | 26.84 (5.32) | | 26.17 (5.25) | | 0.493 | |
| **FEV_1_** *%pred.* | 65.72 (27.84) | | 59.23 (21.89) | | 0.157 | |
| **FEV_1_ / VC** | 52.27 (13.46) | | 53.02 (13.22) | | 0.808 | |
| **Disease severity (GOLD)** |  | |  | | 0.568 | |
| N-Missing | 8 | | 3 | |  | |
| I | 24 (29.6%) | | 10 (19.2%) | |  | |
| II | 30 (37.0%) | | 24 (46.2%) | |  | |
| III | 20 (24.7%) | | 13 (25.0%) | |  | |
| IV | 7 (8.6%) | | 5 (9.6%) | |  | |
| **BODE index** | 2.63 (1.87) | | 2.65 (1.93) | | 0.963 | |
| **6MWD** *m* | 459.82 (101.74) | | 445.20 (91.37) | | 0.427 | |
| **6MWD** *%pred.* | 74.92 (17.86) | | 73.45 (15.76) | | 0.647 | |
| **MRC** | 2.45 (1.28) | | 2.47 (1.22) | | 0.937 | |
| **VQ11** | 25.75 (8.02) | | 25.16 (8.78) | | 0.715 | |
| Data are presented as means (SD). BMI: body mass index; FEV_1_: forced expiratory volume in 1 s; VC: vital capacity; 6MWD: 6-min walking distance; %pred: % predicted; MRC: modified Medical Research Council dyspnea score; VQ11: short health-related quality of life questionnaire. Disease severity classified according to the GOLD guidelines: stage I, mild, FEV1 >80% of predicted normal value; stage II, moderate, FEV1 50–79%; stage III, severe, FEV1 30–49%; stage IV, very severe, FEV1 <30%. | | | | | | |

**Table S2.** Uni- and multivariate analyses of 5-year survival probability.

|  | **Univariate analysis** | |  | **Multivariate analysis** | |
| --- | --- | --- | --- | --- | --- |
| **Cofunders** | **HR** | **p-value** |  | **HR** | **p-value** |
| **Group** *PR only* | 2.9 (1.3-6.2) | 0.008 |  | 3.1 (1.0-9.6) | 0.045 |
| **Age** *years* | 1.1 (1-1.1) | < 0.001 |  | - | - |
| **Gender** *male* | 6.1 (2.1-17) | < 0.001 |  | 3.5 (1.2-10.7) | 0.026 |
| **FEV_1_** *(%pred.)* | 0.96 (0.95-0.98) | < 0.001 |  | - | - |
| **FEV_1_ / VC** | 0.95 (0.92-0.97) | < 0.001 |  | 0.95 (0.92-0.99) | 0.009 |
| **Post-PR 6MWD** *m* | 0.99 (0.99-0.99) | < 0.001 |  | 0.99 (0.99-1.0) | < 0.001 |
| Data are presented as HR (95% CI for HR). FEV_1_: forced expiratory volume in 1 s; VC: vital capacity; 6MWD: 6-min walking distance; %pred: % predicted. | | | | | |

# Additional figures

**Figure S1**. *Comparison of maintenance program adherence and response to initial pulmonary rehabilitation program between PR maintenance program responders and non-responders.* A. Maintenance program adherence in non-responders (gray bar) and responders (black bar). B. Post-to-pre-pulmonary rehabilitation delta of 6MWD values in non-responders (gray bar) and responders (black bar).

**Figure S2**. *Relation between the 6-minute walking distance (6MWD) at program entrance and the response to this program for short-term PR and long-term PR maintenance program.* Short-term: Responders were COPD patients with a gain of 6MWD >35 m after PR completion. Long-term: Responder and non-responder groups are set on the basis of trajectory analysis described on Figure 4 and Table 2.
