# Supplementary figures and images for: Efficacy of a long-term pulmonary rehabilitation maintenance program for COPD patients in a real-life setting: a 5-year cohort study
Source: Respir Res. 2021 Mar 10;22:79. doi: 10.1186/s12931-021-01674-3 (PMC7948332; doi:10.1186/s12931-021-01674-3)

**A**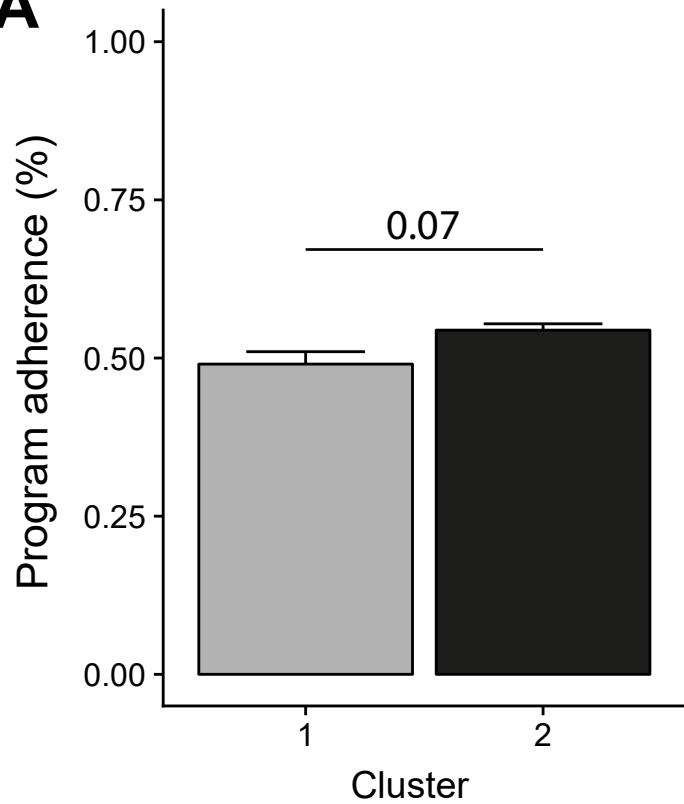**B**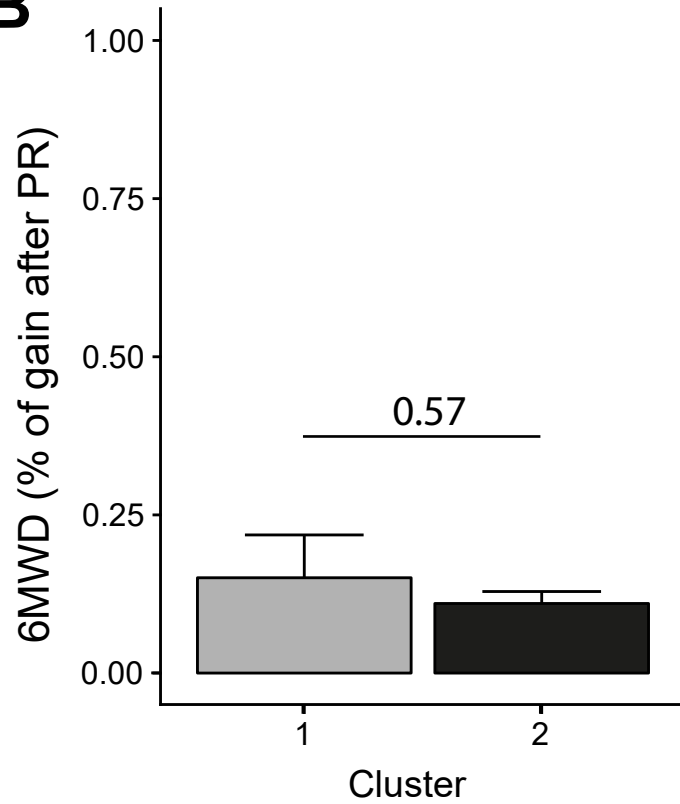

Supplement: Supplementary file 2 — Additional file 2: Fig. S1. Comparison of maintenance program adherence and response to initial pulmonary rehabilitation program between PR maintenance program responders and non-responders. A. Maintenance program adherence in non-responders (gray bar) and responders (black bar). B. Post-to-pre-pulmonary rehabilitation delta of 6MWD values in non-responders (gray bar) and responders (black bar). [file 12931_2021_1674_MOESM2_ESM.pdf]
