# Supplementary material for: Efficacy of a long-term pulmonary rehabilitation maintenance program for COPD patients in a real-life setting: a 5-year cohort study
Source: Respir Res. 2021 Mar 10;22:79. doi: 10.1186/s12931-021-01674-3 (PMC7948332; doi:10.1186/s12931-021-01674-3)
Supplement: Supplementary file 3 — Additional file 3: Fig. S2. Relation between the 6-min walking distance (6MWD) at program entrance and the response to this program for short-term PR and long-term PR maintenance program. Short-term: Responders were COPD patients with a gain of 6MWD > 35 m after PR completion. Long-term: Responder and non-responder groups are set on the basis of trajectory analysis described on Fig. 4 and Table 2. [file 12931_2021_1674_MOESM3_ESM.pdf]

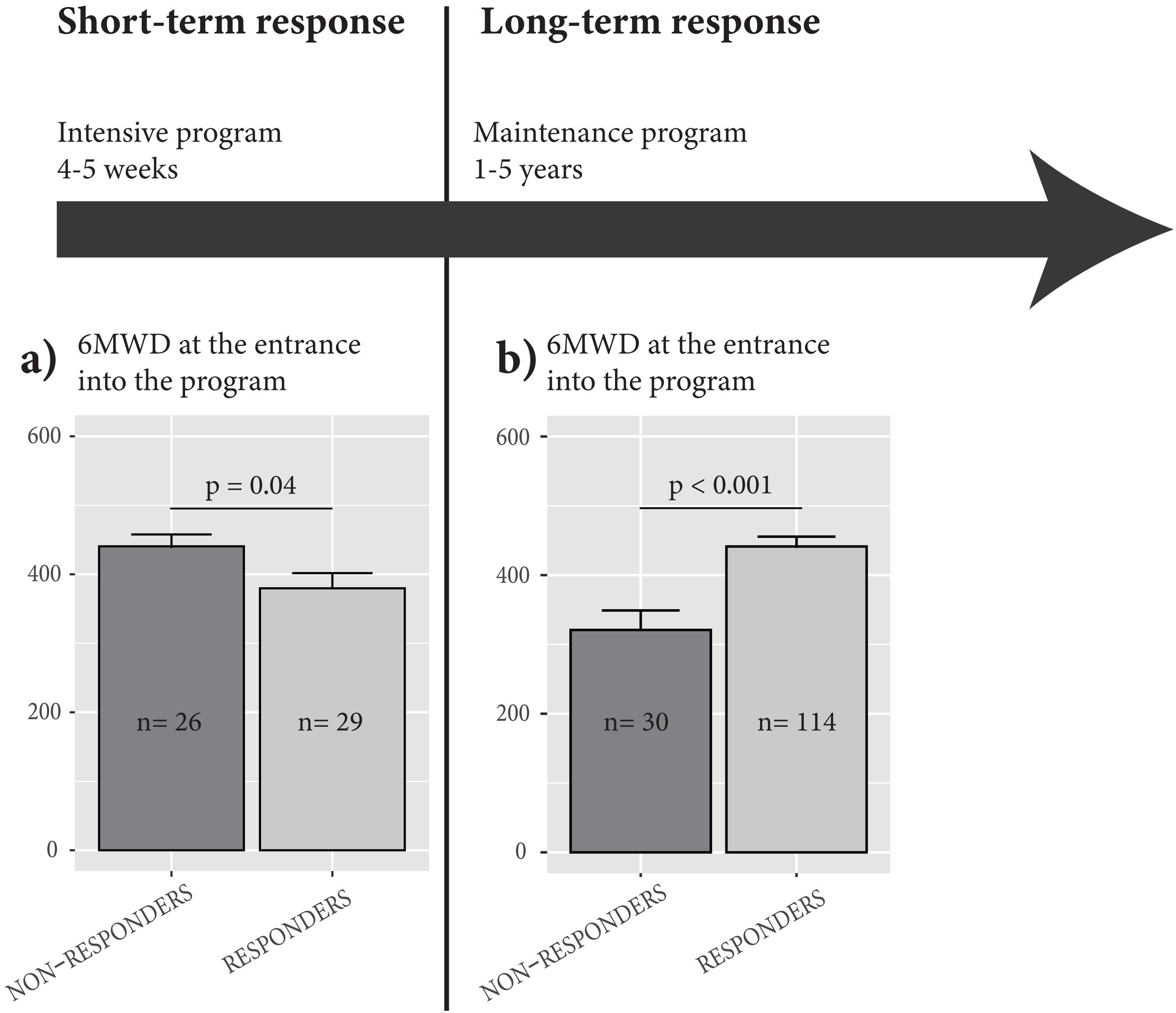
Short-term response
Long-term response
Intensive program
4-5 weeks
Maintenance program
1-5 years
a)
6MWD at the entrance into the program
600
400
200
0
p = 0.04
n= 26
n= 29
NON-RESPONDERS
RESPONDERS
b)
6MWD at the entrance into the program
600
400
200
0
p < 0.001
n= 30
n= 114
NON-RESPONDERS
RESPONDERS
